# Isolation and Characterization of Basidiomycetous Yeasts Capable of Producing Phytase under Oligotrophic Conditions

**DOI:** 10.3390/microorganisms10112182

**Published:** 2022-11-03

**Authors:** Akino Kurosawa, Ryo Nishioka, Nobuhiro Aburai, Katsuhiko Fujii

**Affiliations:** Department of Chemistry and Life Science, Kogakuin University, 2665-1 Nakano-cho, Tokyo 1920015, Japan

**Keywords:** Basidiomycetous yeast, phytase, oligotroph, *Saitozyma*, *Leucosporidium*, *Malassezia*

## Abstract

Phytic acid is an organic phosphorus source naturally produced by plants as phosphorus stock and can be an alternative to rock phosphate, which is a dwindling resource globally. However, phytic acid is insoluble, owing to its binding to divalent metals and is, thus, not readily bioavailable for plants and monogastric livestock. Therefore, the enzyme phytase is indispensable for hydrolyzing phytic acid to liberate free phosphates for nutritional availability, making the screening of novel phytase-producing microbes an attractive research focus to agriculture and animal feed industries. In the present study, a soil-extract-based culture medium was supplemented with phytic acid as the sole phosphorus source and oligotrophic phytase-producing strains, which had not been previously studied, were isolated. Four fungal strains with phytic acid, assimilation activities were isolated. They were found to produce phytase in the culture supernatants and phylogenetic analysis identified three strains as basidiomycetous yeasts (*Saitozyma*, *Leucosporidium*, and *Malassezia*) and one strain as an ascomycetous fungus (*Chaetocapnodium*). The optimal pH for phytase activity of the strains was 6.0–7.0, suggesting that they are suitable for industrial applications as feed supplements or fertilizer additives for farmland.

## 1. Introduction

Phosphorus is a vital component in biomolecules, such as nucleic acids, ATP, and phospholipids, and is, thus, essential for all organisms, with its industrial applications ranging from the production of fertilizers, food additives, and feed additives, to pharmaceuticals. However, due to these diverse applications of phosphorus, the worldwide demand for phosphate rock, the main natural source of phosphorus, has been growing rapidly. Global phosphorus production is projected to peak by 2033 and phosphate rock could be depleted within 100 years [1]. Therefore, it is imperative that new phosphorus sources and recovery technologies be developed to contribute to a sustainable society.

Most recent studies have focused on technologies for phosphate recovery from wastewater and sewage sludge [2]. Apart from these sources, natural soil is also a phosphorus sink, containing approximately 0.5–2.5 g total phosphorus per kilogram of soil [3,4]. However, more than 90% of total phosphorus is in an insoluble form that exists as either metal salts of inorganic phosphate or as phytic acid (PA). While metal (mainly Ca^2+^, Fe^2+^, or Al^3+^) salts of inorganic phosphate are derived from apatite in soil, PA is a six-fold dihydrogen phosphate ester of inositol and the storage form of phosphorus in plant seeds, which becomes insoluble when chelating with divalent metal ions, such as Mg^2+^, Ca^2+^, and Zn^2+^ [5,6]. Therefore, these phosphate compounds are not readily bioavailable to plants and can only be taken up following solubilization by phosphate-solubilizing microbes (PSMs). PSMs can be classified into two groups, namely, acid-secreting strains and phytase-producing strains, based on their mode of action in targeting phosphorus substrates. Specifically, the former solubilizes inorganic phosphate metal salts by secreting organic acids, including oxalic acid, citric acid, and low-molecular-weight fatty acids [7], while the latter metabolizes PA using phytase, a phosphatase that catalyzes the release of inorganic phosphate from PA by hydrolysis [8,9].

Since PA is an abundant and renewable phosphorus compound produced by plants, and phytase is a valuable enzyme used in the production of animal feed and fertilizer [10], novel phytase-producing strains make an attractive research target. While many bacterial and fungal phytase producers have been isolated and characterized to date [8,9], culture-independent metagenomic studies have suggested that the majority of microbes in nature have not been cultivated [11,12] and many phytase-producing strains remain uncharacterized.

To isolate PSMs, Pikovaskaya’s medium, Reyes’s medium, or modified versions of these media are generally employed because they contain insoluble phosphate as the phosphorus source. Colonies of PSMs form a halo zone on these media as the insoluble phosphate is solubilized [13,14,15]. The media are synthetic and contain 10 g L^−1^ of sugar and 0.5 g L^−1^ yeast extract, suitable for isolating copiotrophic PSMs but not oligotrophic PSMs. It is worth noting that natural soil is an environment that contains a lower nutrition level than synthetic media. Therefore, to explore novel oligotrophic phytase producers, a culture medium containing soil constituents may prove useful. The present study aimed to isolate oligotrophic phytase-producing strains by employing a culture medium with a nutritional composition similar to that of natural soil.

## 2. Materials and Methods

### 2.1. Chemicals and Materials

PA (PA calcium salt; CAS 3615-82-5) was purchased from Tokyo Kasei (Tokyo, Japan). Microbial media were obtained from Becton Dickinson and Company (Franklin Lakes, NJ, USA). The reagents for molecular biology were purchased from Toyobo (Osaka, Japan) and Thermo Fisher Scientific (Waltham, MA, USA). Andosol from the Kanto Plain is commercially available from Tachikawa Heiwa Noen (Tochigi, Japan). All other chemicals and materials were purchased from Wako Pure Chemicals (Kyoto, Japan), Merck (Darmstadt, Germany), or Sartorius (Göttingen, Germany).

### 2.2. Preparation of Soil Extract

To prepare the soil extract, 333 g of andosol was suspended in 500 mL of either distilled water, 10 mM CaCl_2_, 100 mM CaCl_2_, or 1.0 M CaCl_2_ and was left to settle at 25 °C for 24 h or autoclaved at 121 °C for 20 min. Each soil suspension was subsequently centrifuged at 6000× *g* for 10 min at 25 °C and the supernatant was filtered through Whatman No. 2 filter paper. The pH of the filtrate was adjusted to 7.0 using 1.0 M NaOH. The total organic carbon and nitrogen content of each soil extract was determined using a Shimadzu TOC-VCSH total organic carbon analyzer (the combustion catalytic oxidation/NDIR method) equipped with a TNM-1 total nitrogen measuring unit (the oxidative combustion/chemiluminescence method). Phosphorus content of the soil extract was determined using the molybdenum blue method developed by Murphy and Riley [16].

### 2.3. Preparation of Culture Media

In the present study, four media, namely, SP, SCP, YMG, and YMP, were used to cultivate the isolates. Filtrate of autoclaved soil–water extract supplemented with 100 mg L^−1^ glucose and 10 mg L^−1^ PA constituted the SP medium, while filtrate of autoclaved soil–1.0 M CaCl_2_ extract supplemented with 100 mg L^−1^ glucose and 10 mg L^−1^ PA constituted the SCP medium. YMG medium contained 10.0 g L^−1^ glucose, 5.0 g L^−1^ peptone, 3.0 g L^−1^ malt extract, and 3.0 g L^−1^ yeast extract, while YMP medium contained 10.0 g L^−1^ PA instead of glucose, 5.0 g L^−1^ peptone, 3.0 g L^−1^ malt extract, and 3.0 g L^−1^ yeast extract. For solid cultivation of the isolates, the abovementioned liquid media solidified with 20.0 g L^−1^ Bacto agar were used.

### 2.4. Sampling of Soils and Isolation of PA-Solubilizing Microbes

To isolate PA-solubilizing microbes, three soil samples were collected at Inariyama, Tanakayama, and Kitahara metropolitan parks located in Tokyo and were screened through a 2 mm sieve. The sieved soil sample (5 g) was suspended in 30 mL of sterile water and vortexed vigorously for 1 min. The resultant soil suspension (100 μL) was inoculated into 20 mL of the SCP medium and cultivated for two weeks at 30 °C with constant shaking at 120 rpm. The cultures were subcultured three times and aliquots of their successive subcultures (20 μL each) were streaked onto the SCP medium solidified with 20.0 g L^−1^ agar and then incubated for 1 week at 30 °C to isolate the microbes. Emerging single colonies were examined for further studies, as described below. Plate count agar (10.0 g L^−1^ glucose, 5.0 g L^−1^ tryptone, 2.5 g L^−1^ yeast extract, and 20.0 g L^−1^ agar) was used to determine the colony-forming units (CFUs) of total culturable microbes in the soil samples.

### 2.5. Phytase Assay

The isolates were cultivated in 60 mL of SP, SCP, YMG, and YMP medium for one week at 30 °C with constant shaking at 120 rpm. The supernatants from the 1-week culture of each isolate were separated from fungal cells by centrifugation at 6000× *g* for 10 min and then filtered through a Merck 0.2 μm Omnipore filter (Merck, NJ, USA). The filtrate (50 mL) was subjected to a Sartorius VIVASPIN 20 ultrafiltration unit (MWCO 10,000 Da) and the resultant filter residue was resuspended in distilled water (1.5 mL) to obtain a concentrated enzyme solution.

To determine the activity of phytase from the culture supernatant of the isolates, 80 μL of the concentrated enzyme solution was added to 3.0 mL of 100 mM HEPES-NaOH buffer (pH 7.0) containing 0.1% (*w*/*v*) of PA and 0.01% (*w*/*v*) of sodium azide and the mixture was incubated at 30 °C for five days. The five-day reaction mixture was centrifuged at 10,000× *g* for 10 min at 4 °C to recover the supernatant and the soluble phosphate concentration was determined using the molybdenum blue method. To determine phytase activity at pH 5.0, pH 6.0, and 8.0, 100 mM acetate buffer (pH 5.0), 100 mM MES-NaOH buffer (pH 6.0), and 100 mM HEPES-NaOH buffer (pH 8.0) were used, respectively. One enzyme unit (U) of phytase was defined as the amount of enzyme that liberates 1 nmol of phosphate in 1 min [17]. Protein concentration in the culture supernatant was determined using the bicinchoninic acid method [18]. Statistical analysis of enzyme activity was performed using Student’s *t*-test.

### 2.6. Phylogenetic Analysis of the Isolated Strains

Cells of the strains grown on agar medium were collected into sterile microtubes using disposable inoculating loops and suspended in 300 μL of 1.0% Triton X100-TE buffer (pH 8.0). The cell suspension was then mixed with 200 mg of glass beads (1.0 mm diameter) and zirconia beads (0.5 mm diameter) and was homogenized in BHA-6 bead-beating homogenizer (AS ONE, Tokyo, Japan) at 4350 rpm for 1 min, heated in boiling water for 10 min, and chilled on ice. The resulting cell lysate was subjected to phenol–chloroform extraction and the aqueous layer was recovered, which contained the DNA to be used as PCR template. PCR was performed to amplify the internal transcribed spacer (ITS) regions (approximately 500 base pairs (bp) in length, including ITS1, 5.8S, and ITS2 regions) of fungal ribosomal DNA using KOD One DNA polymerase (Toyobo) and the primers ITS1 (5′-TCCGTAGGTGAACCTGCGG-3′) and ITS4 (5′-TCCTCCGCTTATTGATATGC-3′), as described by White et al. [19]. The PCR step consisted of 30 cycles of reactions being run at 98 °C for 10 s, 54 °C for 30 s, and 68 °C for 1 min. The PCR products were purified using a GeneJET PCR Purification Kit (Thermo Fisher Scientific) and stored at −30 °C until sequencing.

Direct sequencing of the amplified DNA fragments was performed using a BigDye Terminator v3.1 (Thermo Fisher Scientific) and similarities of the sequences to known species were evaluated by comparing them with the sequence data in the GenBank, EMBL, and DDBJ databases using the BLAST algorithm. Phylogenetic trees were constructed using the maximum likelihood method in MEGA6 program.

## 3. Results

### 3.1. Development of Soluble-Phosphate-Free Soil Extract

**Figure 1:**
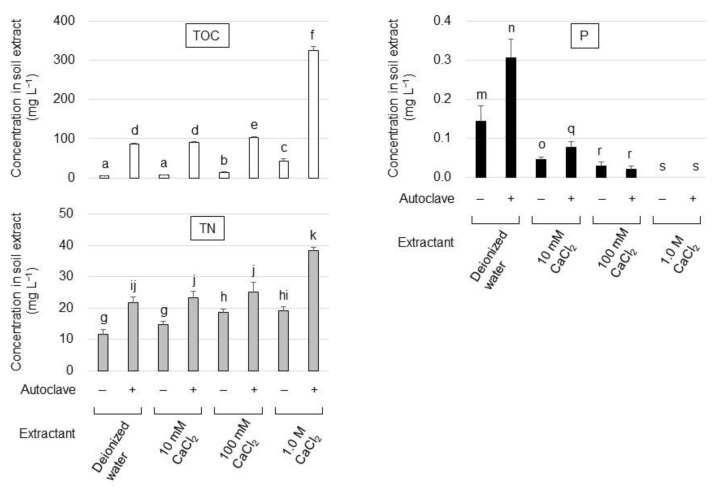
Total organic carbon (TOC), total nitrogen (TN), and phosphorus (P) concentration in the soil extract under different extraction conditions. Symbols + and—indicate that the extract was obtained with or without autoclave treatment, respectively. The data are presented as means ± standard deviation of independent triplicates. Columns with different letters (a–k, m–o, q–s) indicate significant difference at *p* < 0.05 (Student’s *t*-test).

To develop a culture medium suitable for the cultivation of oligotrophic phytase-producing strains, soil extracts that contained a carbon and nitrogen source but were free of soluble phosphates were prepared. The andosol extract was prepared under various treatment conditions (Figure 1). Autoclaves were found capable of extracting organic carbon, nitrogen, and phosphate more efficiently than room-temperature extraction. As the absence of soluble phosphates in the medium is ideal for isolating phytase-producing strains, the effect of CaCl_2_ on the immobilization of soluble phosphates in the soil extract was examined and 1.0 M CaCl_2_ was found to be essential for a complete precipitation of soluble phosphates and efficient carbon and nitrogen extraction from soil. Maximal values for total organic carbon (TOC) (325 ± 11.2 mg L^−1^) and total nitrogen (TN) (38.5 ± 1.06 mg L^−1^) were obtained from the soil extract prepared with 1.0 M CaCl_2_ at 121 °C for 20 min (autoclaved soil–1.0 M CaCl_2_ extract) and the resulting TOC/TN ratio was 8.43. Therefore, the filtrate of the autoclaved soil–1.0 M CaCl_2_ extract was neutralized to pH 7.0 by adding NaOH, supplemented with 100 mg L^−1^ glucose and 10 mg L^−1^ PA, which was then solidified with agar to be used as a solid medium (termed SCP medium henceforth) for the isolation of PA-hydrolyzing strains.

### 3.2. Isolation of PA-Hydrolyzing Microbes

**Figure 2:**
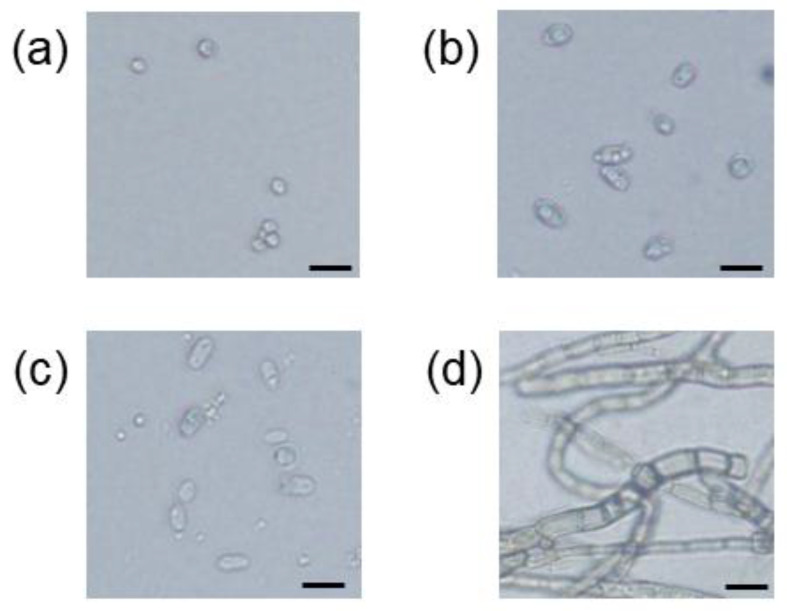
Optical micrographs of cells of the strain I1 (**a**), strain KW1 (**b**), strain TW1 (**c**), and strain TG1 (**d**). Black scale bar = 20 μm.

The number of total culturable microbes in the soil samples from Inariyama, Tanakayama, and Kitahara metropolitan parks, when plated on plate count agar, was 2.0 × 10^6^ CFU/g dry soil, 4.0 × 10^6^ CFU/g dry soil, and 2.4 × 10^6^ CFU/g dry soil, respectively. To isolate PA-hydrolyzing microbes present in the soil samples, aliquots of third successive enrichment culture of the soil samples were inoculated onto SCP agar medium. After one week of cultivation, microbial colonies emerged on the agar medium. For the soil samples from the Inariyama, Kitahara, and Tanakayama parks, the concentrations of microbes grown on SCP agar medium were 2400 CFU/g dry soil, 1400 CFU/g dry soil, and 1800 CFU/g-dry soil, respectively. Colonies on agar medium inoculated with subculture of soil suspension from the Inariyama and Kitahara parks were morphologically identical, while white and grey colonies were observed on agar medium inoculated with subculture of soil suspension from the Tanakayama Park. Therefore, based on morphology, four colonies were isolated and designated: strain I1 from the Inariyama Park soil, strain KW1 from the Kitahara Park soil, and two strains, TG1 and TW1, from the Tanakayama Park soil. Microscopic observation revealed that strain TG1 (Figure 2d) was a fungal strain possessing long hyphae with 4.5–11 μm width, while cells of the strains I1 (Figure 2a), KW1 (Figure 2b), and TW1 (Figure 2c) possess yeast cell-like ovoid shape with cell lengths of 6.7–9.3 μm, 11–19 μm, and 9.3–16 μm in long axis, respectively.

### 3.3. Phytase Activity of the Isolated Strains

**Figure 3:**
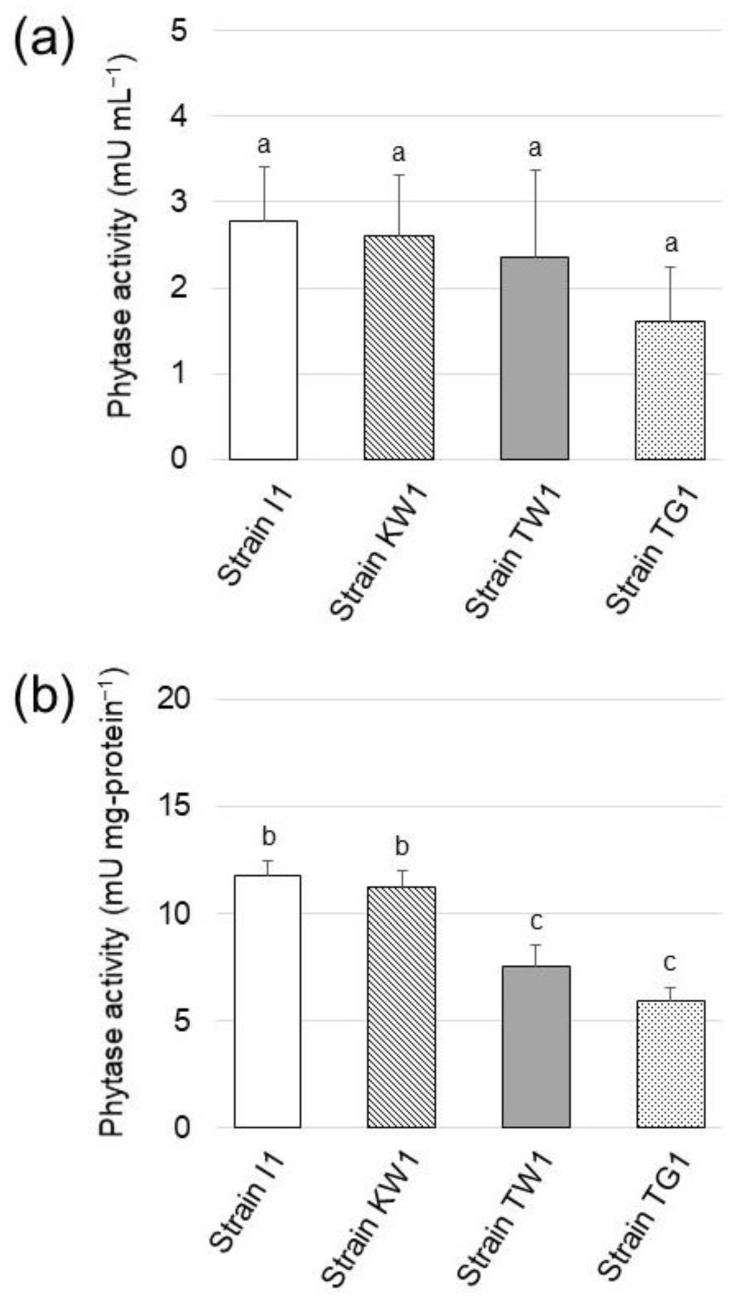
(**a**) Phytase activity detected in the culture supernatant of the isolated strains grown in SCP medium (pH 7.0). (**b**) Specific enzyme activity of phytase in the isolated strains. The data are presented as means ± standard deviation of independent triplicates. Columns with different letters (a–c) indicate significant difference at *p* < 0.05 (Student’s *t*-test).

Phytase activity of the isolated strains was examined. Figure 3a shows the phytase activity in the supernatant of the 1-week culture for each strain. All the strains were found to possess phytase activity ranging from 2.79 to 1.61 mU mL^−1^, but statistical analysis (*t*-test) revealed that there were no significant differences in the phytase activity of the isolates. In contrast, significant differences were found in the specific enzyme activity among the isolates (Figure 3b), with the highest activity exhibited by strain I1 and strain KW1 phytases (11.8 and 11.3 mU per mg-protein, respectively).

**Figure 4:**
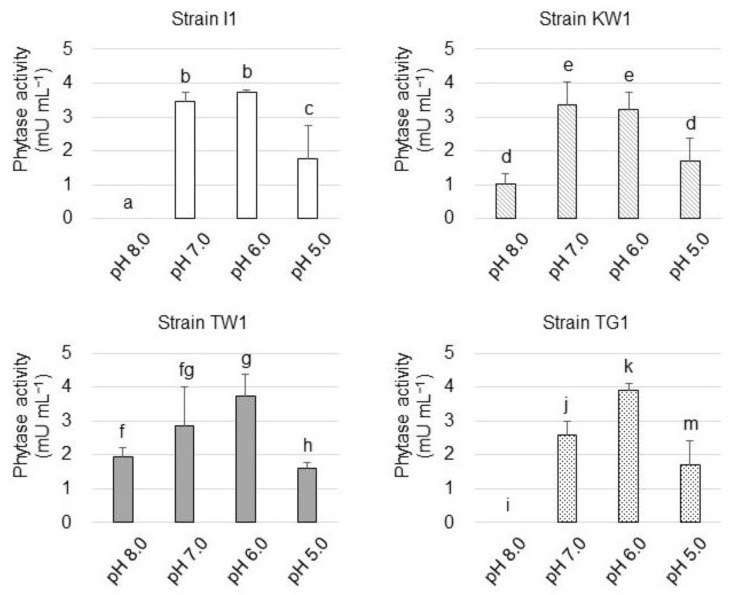
Phytase activity of the isolated strains at different pH values. The data are presented as means ± standard deviation of independent triplicates. Columns with different letters (a–k and m) indicate significant difference at *p* < 0.05 (Student’s *t*-test).

Subsequently, the influence of pH on the phytase activity was examined (Figure 4). Strain I1 phytase hydrolyzed PA at pH 6.0 and 7.0, but its activity was partly lost at pH 5.0 and completely lost at pH 8.0. The activity of strain KW1 phytase exhibited a trend similar to that of the strain I1 phytase, but unlike strain I1 phytase, KW1 phytase remained weakly active at pH 8.0. As for strain TW1 and TG1 phytase, the highest enzyme activity was observed at pH 6.0, but the activity was reduced at pH 5.0 and pH 8.0.

### 3.4. Phylogenetic Classification of the Isolated Strains

**Figure 5:**
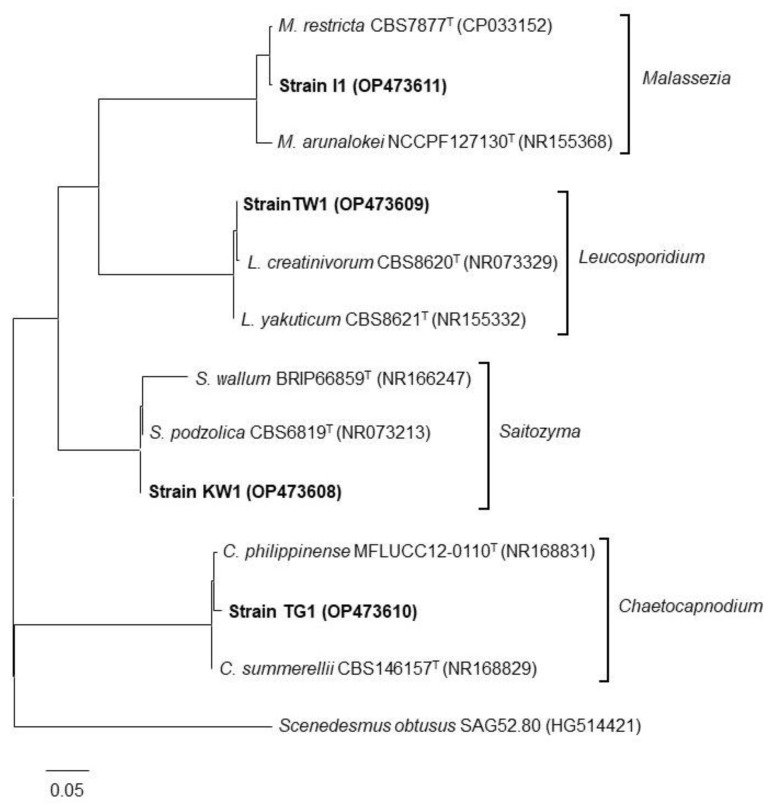
Phylogenetic tree of the isolated strains (indicated in bold) with its known neighbors based on ITS sequences constructed using the maximum-likelihood method. The scale bar represents an evolutionary distance (Knuc) of 0.05. Accession numbers are indicated in parentheses for each DNA sequence.

The phylogenetic positions of the isolated strains were determined based on the ITS region sequences. Figure 5 shows the phylogenetic tree for the strains and their closest known relatives, as constructed using the maximum-likelihood method. The strains were classified into four genera: *Saitozyma* (basidiomycetous yeast), *Leucosporidium* (basidiomycetous yeast), *Malassezia* (basidiomycetous yeast), and *Chaetocapnodium* (ascomycetous fungi). DNA sequence similarities in the ITS region between strain KW1 and *Saitozyma podzolica*, strain I1 and *Malassezia restricta*, strain TW1 and *Leucosporidium yakuticum*, as well as strain TG1 and *Chaetocapnodium summerellii,* were 98.6%, 99.6%, 99.8%, and 98.0%, respectively.

### 3.5. Phytase Production Mode of the Isolated Strains

**Figure 6:**
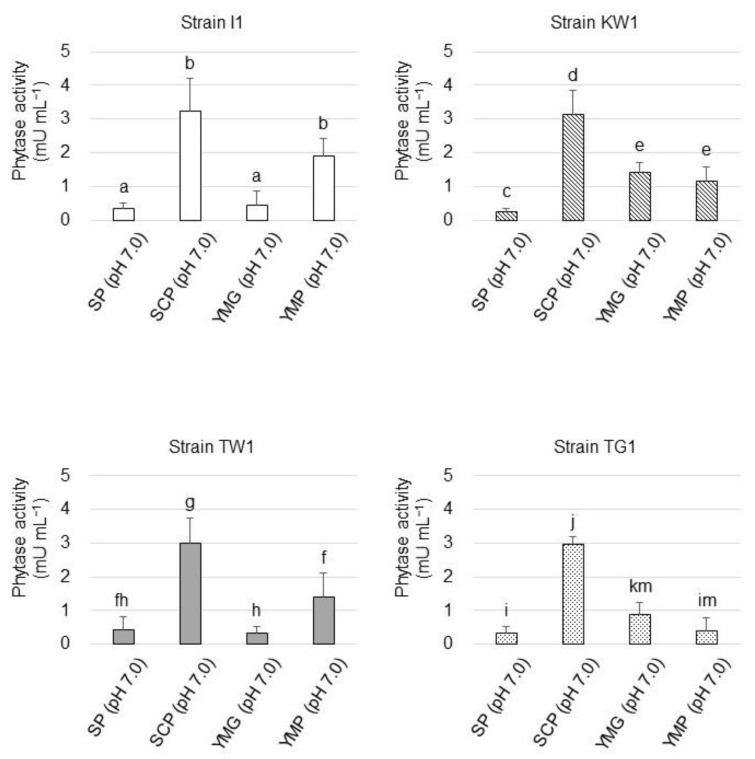
Phytase yield of the isolated strains grown at different nutritive levels based on the enzyme activity in their culture supernatants. The data are presented as means ± standard deviation of independent triplicates. Columns with different letters (a–k and m) indicate significant difference at *p* < 0.05 (Student’s *t*-test).

The influence of the nutritive level in the culture medium, including SP (pH 7.0), SCP (pH 7.0), YMG (pH 7.0), and YMP (pH 7.0) media, on phytase production by the strains was studied (Figure 6). Phytase yield appeared to be the highest in all the strains when they were cultivated in the SCP medium, which was composed of autoclaved soil–1.0 M CaCl_2_ extract, supplemented with glucose and PA as the sole phosphorus source. Phytase yield was found to decrease when the isolates were cultivated in SP medium, which was made of autoclaved soil–water extract and, thus, contained soluble soil phosphorus. Phytase production by the isolates was not stimulated when they were cultivated in the copiotrophic medium (YMG and YMP media) in comparison to their phytase production in the SCP medium.

## 4. Discussion

As global phosphorus shortage looms in various industrial fields, including agriculture, livestock, and chemical manufacturing, the exploration of novel phosphorus sources becomes a pressing need for the sustainable development of society. Among phosphorus-containing resources, PA in soil and crop residues remains a potential renewable phosphorus source because it is naturally produced by plants as phosphorus stock using energy produced by photosynthesis. However, most PA in soil chelates metal cations and exists in an insoluble form and economically feasible technologies for PA solubilization remain to be established. Phytase is a beneficial enzyme for PA solubilization as it can liberate phosphates from inositol and, hence, is employed in feed industries to improve nutrition quality of feeds and reduce phosphorus pollution caused by livestock excrement. However, efforts to reduce the production cost of this enzyme are still required to promote a more widespread application. The discovery of new phytase-producing strains, as well as the improvement in the bioactivity of known phytases by genetic engineering, may catalyze such efforts.

The present study attempted to isolate oligotrophic phytase-producing strains that could grow at nutritive levels close to those found in the soil environment. First, a soil-extract-based medium suitable for cultivation of oligotrophic strains, termed SCP medium, was developed. The TOC content of the resulting SCP medium was determined to be 368 mg L^−1^, in contrast to the reported TOC content of Pikovaskaya’s medium and Reyes’s medium, which were 4188 mg L^−1^ [13,20] and 12,620 mg L^−1^ [3], respectively. Additionally, the TOC/TN ratio (8.43) of SCP medium was found to be close to the range of ratios of a typical forest soil (10.9–12.2) [21] and phosphorus immobilized by CaCl_2_ contained in SCP medium was not hydrolyzed by autoclaving, suggesting that the SCP medium contained a nutritive level that is suitable for the cultivation of oligotrophic soil microbes.

Aliquots of the soil suspension from each sampling location were cultivated on copiotrophic agar medium (plate count agar) and SCP agar medium (oligotrophic agar) to estimate the abundance of culturable microbes and phytase-producing strains, respectively. The CFU values from both agar media suggested that only 0.12%, 0.035%, and 0.075% of total culturable microbes in soil samples of Inariyama, Tanakayama, and Kitahara metropolitan parks, respectively, could grow by assimilating PA as the sole phosphorus source. Phytase activity was detected in the culture supernatant of all strains and a comparison of their specific enzyme activity suggested that strains I1 and KW1 possessed the most potent phytase.

From the phylogenetic analysis, three strains belonged to basidiomycetous yeast genera (*Malassezia*, *Leucosporidium*, and *Saitozyma*) and one strain belonged to ascomycetous fungi (*Chaetocapnodium*). Cells of those basidiomycetous yeast genera or ascomycetous fungi genus are known to form ovoid shapes [22,23,24] or hyphae [25], respectively, which is consistent with microscopic observation in the present study. Several studies have been published on phytase-producing bacterial and fungal strains, including ascomycetous yeasts [26]; however, there are no reports on the phytase activity of basidiomycetous yeasts. Therefore, our study is the first to report on the phytase activity of these yeasts. Phytase activity in the culture supernatant of known ascomycetous yeast strains is reported to be within a range of 1–92 mU mL^−1^ [27], which is comparable to the phytase activity observed in our strains. Conversely, specific enzyme activity for phytase of the strains is lower than that for *Aspergillus oryzae* (500 mU mg-protein^−1^), a commercialized phytase producer [28]. In contrast to the yeast phytase assay in published works, which employed sodium PA (soluble PA) as a substrate, our strains solubilized insoluble PA calcium salt. As PA is bound to metal ions in natural environments and remains in an insoluble form, phytase strains reported in this study may be better suited for application in agriculture and the feed industry. *Leucosporidium* is an obligate psychrophilic yeast [29]. The genus contains 13 species and *L. creatinivorum*, the closest known relative of strain TW1, has been reported to be an oleaginous species [30]. *Saitozyma* encompasses six soil-inhabiting species and *S. podozolica*, the closest relative of strain KW1, is also an oleaginous species [31]. *Malassezia* is a genus of lipolytic yeasts associated with the skin of animals, including humans, and some species can cause dermatitis [32]. Remarkably, the closest relatives of the yeast isolates in this study exhibit metabolisms related to lipids (lipid accumulation/degradation), although we could not find reasons for the correlation between lipid metabolism and phytase activity.

Phytase is classified as either acid phytase or alkaline phytase [33] based on its optimal pH value. Since the phytase activity of all strains was reduced at pH 8.0, the phytase they produced is likely to be acid phytase.

The mode of phytase production by the strains was also studied. The phytase yield of all the strains cultivated in the SP medium remained low in comparison to that in the SCP medium, suggesting that the presence of insoluble phosphorus substrate (PA in this study) and a lack of soluble phosphorus source are essential for phytase gene expression. Phytase production was accelerated when strains I1 and TW1 were cultivated in YMP compared to YMG. While YMG medium contains only soluble phosphates as the phosphorus source, PA serves as both the phosphorus source and the major carbon source in the YMP medium. Thus, excess PA could stimulate phytase gene expression in strains I1 and TW1.

The employment of phytase from oligotrophic strains might reduce enzyme production cost as they demand fewer nutritional requirements to grow than most bacteria and fungi, as suggested by the strains in the present study. Considering that the culture medium for enrichment and isolation of the strains was composed mainly of soil extract, we expect that the strains might survive and produce phytase under soil conditions. The optimal pH for phytase activity of the strains was 6.0–7.0, suggesting that they are suitable as fertilizer additives for farmland or feed supplements, because pH values for general farmland soils and animal feeds range from 6 to 7 [34,35,36]. However, in order to conclude availability of the strains for industrial application as PA solubilizers or phytase producers, a proof-of-concept study is indispensable. It should be noted that more efforts are needed to develop a practical technology for the application of the strains, which currently is the focus of our subsequent studies.

## Data Availability

The data presented in this study are available on request from the corresponding author.
